# Adsorption of Magenta Dye on PbO Doped MgZnO: Interpretation of Statistical Physics Parameters Using Double-Layer Models

**DOI:** 10.3390/ijerph191912199

**Published:** 2022-09-26

**Authors:** Tariq Altalhi, Ganesh Jethave, Umesh Fegade, Gaber A. M. Mersal, Mohamed M. Ibrahim, M.H.H. Mahmoud, Tushar Kumeria, Kalpesh A. Isai, Milind Sonawane

**Affiliations:** 1 Department of Chemistry, College of Science, Taif University, P.O. Box 11099, Taif 21944, Saudi Arabia; 2 Department of Chemistry, Dr. Annasaheb G. D. Bendale Mahila Mahavidyalaya, Jalgaon 425001, Maharashtra, India; 3 Department of Chemistry, Bhusawal Arts, Science and P. O. Nahata Commerce College, Bhusawal 425201, Maharashtra, India; 4 School of Materials Science and Engineering, The University of New South Wales, Sydney, NSW 2052, Australia; 5 Department of Applied Science and Humanities, R. C. Patel Institute of Technology, Shirpur 425405, Maharashtra, India

**Keywords:** PbO@MgZnO, Magenta Dye, statistical physics models, multi-layer adsorption, reuse study

## Abstract

This article reports the synthesis of PbO doped MgZnO (PbO@MgZnO) by a co-precipitation method, followed by an ultrasonication process. PbO@MgZnO demonstrates a significant adsorption capability toward Magenta Dye (MD). The greatest adsorption capability was optimized by varying parameters such as pH, MD concentration, and adsorbent dose. The kinetics study illustrates that the adsorption of MD on PbO@MgZnO follows the pseudo-second-order. The isotherm study revealed that Langmuir is best fitted for the adsorption, but with little difference in the R^2^ value of Langmuir and Freundlich, the adsorption process cloud be single or multi-layer. The maximum adsorption capacity was found to be 333.33 mg/g. The negative ΔG refers to the spontaneity of MD adsorption on PbO@MgZnO. The steric parameters from statistical physics models also favor the multi-layer adsorption mechanism. As a function of solution temperature, the parameter *n* pattern has values of *n* = 0.395, 0.290, and 0.280 for 298, 308, and 318 K, respectively (i.e., all values were below 1). Therefore, horizontal molecule positioning and multiple locking mechanisms were implicated during interactions between MD and PbO@MgZnO active sites.

## 1. Introduction

Dye pollution is a major organic hazard that is faced by the world [1,2,3]. The production and use of dye are increasing day by day [4]. Dye is used as a coloring agent in many industries, but its dark side is its carcinogenic nature; long-term inhalation causes lung cancer [5,6,7]. Due to the adverse impact of dye on humans as well as ecosystems, scientists are trying to develop new methods to easily remove dye from liquid or wastewater [8,9,10,11].

In addition to being esthetically objectionable, releasing the dye waste products into the surrounding atmosphere impedes the transfer of solar light to receiving water bodies, thereby reducing the dissolved oxygen content and photosynthesis [12,13,14]. These can induce inflammation of the skin, dermatitis, allergy, cancer, or genetic mutations in humans. Dye is not biodegradable [15,16,17,18]. Ecological disaster results from the discharge of certain colorful effluents without efficient treatment. Thus, an important activity is to decolorize dye-laden effluents before releasing them into natural streams [19,20,21,22,23,24,25].

The adsorption approach is very successful among different techniques of color removal. This procedure is usually used in pretreatment or specialized wastewater treatment [26,27,28]. The ability of the dyeing depends, however, on the form of sorbent. Finding an adsorbent with a high adsorbent potential that is readily accessible, is, therefore, an enormous task. Moreover, lately, much attention has been paid to the sorbing products derived from agricultural or industrial waste [29,30,31].

In the last few years, we have synthesized metal ion sensors and fabricated a few metal oxides to solve some environmental issues like water pollution, alternative for fuels and energy, etcetera [32,33,34,35,36,37,38,39,40,41,42,43]. In the current article, we synthesized PbO@MgZnO and used it for the adsorption of MD dye. The MD adsorption, kinetic order, thermodynamic, and multilayer models with the statistical interpretation of various parameters and their significance are also studied. The study also makes the bridge between the experimental and theoretical models (kinetic, isotherm, and multilayer models).

## 2. Experimental

### 2.1. Chemicals and Materials

All chemicals for the synthesis of nanomaterials and application experiments are of analytical grade and are obtained by Fisher Scientific. Mili Q water was used to produce the stock and working solution for the experiments.

### 2.2. Preparation of PbO@MgZnO

The three solutions of Pb(CH_3_COO)_2_, MgNO_3_, and ZnCl_2_ (gm added) in the ratio of 0.02:0.1:1 were prepared. 1 mL of tween 80 is mixed in 25 mL of water and stirred till proper mixing. In the next step, Pb(CH_3_COO)_2_, MgNO_3_, ZnCl_2_, and tween 80 solutions are mixed and stirred at 50 °C for 30 min. After that, the solution is transferred in an ultrasonication bath, with the temperature fixed at 40 °C. 10 N NaOH solutions are prepared and dropwise added in the above solution and sonicated for 1.5 h. The precipitation occurred in the solution and the solution become viscous. The solution is filtered and washed using distilled water and dried in the oven at 120 °C for 5 h and calcinated in a muffle furnace at 450 °C for up to 3 h.

### 2.3. Characterization

The FE-SEM was used to (Make-Bruker Modal-S-4800) observe the morphology of the PbO@MgZnO with a driving voltage of 15 kV. The concentration of each molecule in the metal oxide nanoparticle is illustrated by the EDAX scan on PM picture dimension: 500 × 375 Mag: 40,000 × HV: 15.0 kV. Functional group analysis was done on the FTIR range of 500–4000 cm^−1^ (Model: FT-IR Bruker).

### 2.4. Mathematical Modeling for the MD Adsorption

The use of conventional models, however, has led to a narrow description of the absorption process, because the models’ parameters are analytical and do not have physical correlates. This model has been built based on basic assumptions for processes of adsorption and/or interactions between adsorption and adsorbate, thus resulting in an imperfect interpretation of color extraction, technically. An advanced multi-layer model (generalized model) was used here to attempt a theoretical interpretation of the MD molecule or adsorption process. Please notice that this model has properties parameters like the physical meaning of the phase of adsorption. Contrary to the overall assumption of Langmuir, the model of statistic physics suggests that a fixed number (which may be equal, less, or greater than 1) is the major active site, as the active MD-dye molecules site. This parameter will then provide function knowledge. Data modeling is presumed to be based on the assimilation of a certain number of layers of the dye molecules on the surface of PbO@MgZnO, which is dependent on the absorption temperature. This multi-layer source has surface adsorption energy: MD-PbO@MgZnO interacts with surface adsorption energy, and MD-MD dye molecule interaction, which has second surface adsorption energy. The following mathematical model explains the difference between the amounts of adsorbed MD dye:

Monolayer Model 1:

$$Q_{a}=\frac{nN_{M}}{1+{\left(\frac{C_{1/2}}{C}\right)}^{n}}$$

Double Layer Model 2:

$$Q=n.N_{M}\frac{{\left(\frac{C}{C_{1}}\right)}^{n}+2{\left(\frac{C}{C_{2}}\right)}^{2n}}{1+{\left(\frac{C}{C_{1}}\right)}^{n}+{\left(\frac{C}{C_{2}}\right)}^{2n}}$$

Multilayer Model 3:

$$Q_{a}\;=\frac{\left[n{*N}_{M}*\frac{-2{\left(\frac{C}{C_{1}}\right)}^{2n}}{\left(1\;-\;{\left(\frac{C}{C_{1}}\right)}^{n}\right)}+\frac{{\left(\frac{C}{C_{1}}\right)}^{n}\left(1\;-\;{\left(\frac{C}{C_{1}}\right)}^{2n}\right)}{{\left(1\;-\;{\left(\frac{C}{C_{1}}\right)}^{n}\right)}^{2}}+2\frac{{\left(\frac{C}{C_{1}}\right)}^{n}{\left(\frac{C}{C_{2}}\right)}^{n}\left(1\;-\;{\left(\frac{C}{C_{2}}\right)}^{{nN}_{2}}\right)}{\left(1\;-\;{\left(\frac{C}{C_{2}}\right)}^{n}\right)}\;-\;\frac{{\left(\frac{C}{C_{1}}\right)}^{n}{\left(\frac{C}{C_{2}}\right)}^{n}{\left(\frac{C}{C_{2}}\right)}^{{nN}_{2}}N_{2}\left(1\;-\;{\left(\frac{C}{C_{2}}\right)}^{n}\right)}{\left(1\;-\;{\left(\frac{C}{C_{2}}\right)}^{n}\right)}+\frac{{\left(\frac{C}{C_{1}}\right)}^{n}{\left(\frac{C}{C_{2}}\right)}^{2n}\left(1\;-\;{\left(\frac{C}{C_{2}}\right)}^{{nN}_{2}}\right)}{{\left(1\;-\;{\left(\frac{C}{C_{2}}\right)}^{n}\right)}^{2}}\right]}{\frac{\left(1\;-\;{\left(\frac{C}{C_{1}}\right)}^{2n}\right)}{\left(1\;-\;{\left(\frac{C}{C_{1}}\right)}^{n}\right)}+\frac{{\left(\frac{C}{C_{1}}\right)}^{n}{\left(\frac{C}{C_{2}}\right)}^{n}\left(1\;-\;{\left(\frac{C}{C_{2}}\right)}^{{nN}_{2}}\right)}{\left(1\;-\;{\left(\frac{C}{C_{2}}\right)}^{n}\right)}}$$

The design parameters are as follows: *n* is the number of MD dye molecules adsorbed into the PbO@MgZnO, Nm is receptor site density, C_1_ and C_2_ are quasi-saturation, and N_2_ is the second energy layer with a global formed number of layers equal to 1 + N_2_, respectively. Different adsorption scenarios (1 + N_2_) can be used in this parameter because N_2_ can equal 0, 1, 2, 3, 4, etc. MD adsorption was observed by the parameter value of this model. A certain number of molecular levels (0, 1, 2, 3, etc.) are generated to adsorb the dye on PbO@MgZnO. The parameters of the computational model were calculated using the Levenberg-Marquardt Equation and using non-linear regression of experimental adsorption isotherms, the R^2^ determination coefficient was defined. To choose a single layer, double layer, or three-layer adsorption model, the N_2_ was also set to 0, 1, or 2 during the data fitting process. The test model determination coefficients ranges in between R^2^ 0.997 and 0.998 and thus provide a satisfactory comparison between the experimental results and the model-projected values. A general examination of the model parameters indicates that it is best to describe the adsorption function with the simplified model. This common adsorption model was then potentially used to describe the MD-PbO@MgZnO adsorption process. The following calculations have been used to measure adsorption energies:

(1) $$\varepsilon_{1\;}=\mathrm{RT}\;\ln\;\left(\frac{C_{S}}{C_{1}}\right)$$

(2) $$\varepsilon_{2\;}=\mathrm{RT}\;\ln\;\left(\frac{C_{S}}{C_{2}}\right)$$

## 3. Results and Discussion

### 3.1. Properties of PbO@MgZnO

**Figure 1:**
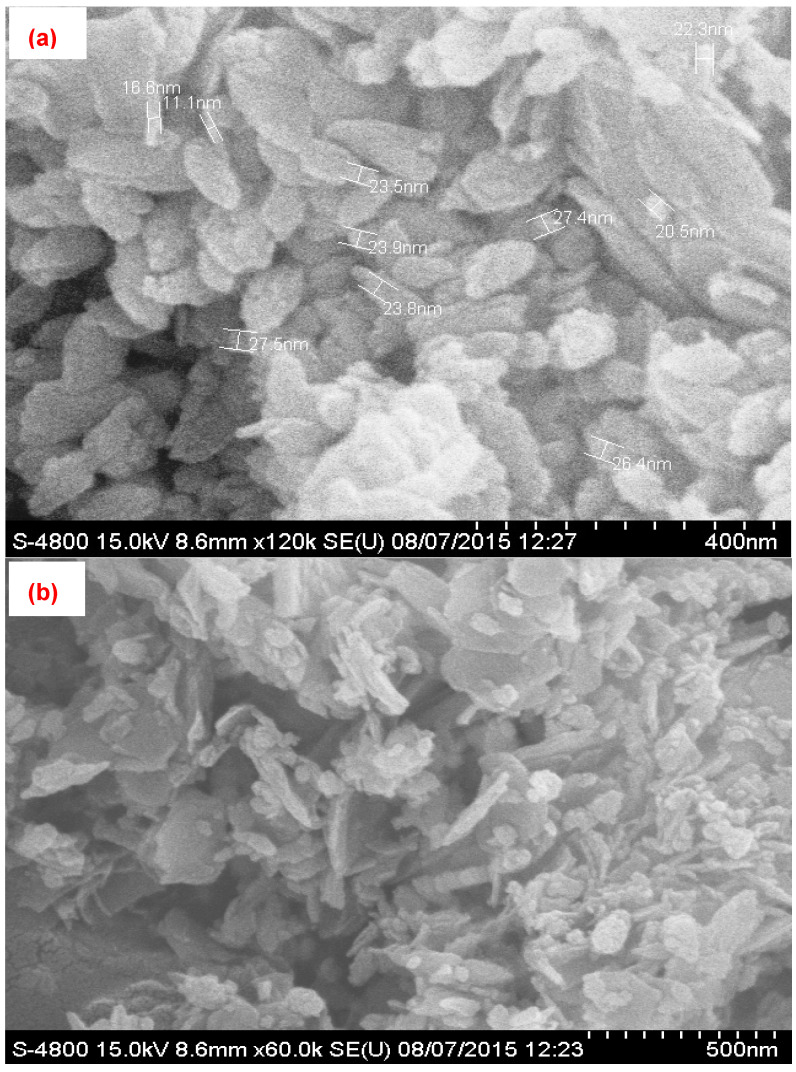

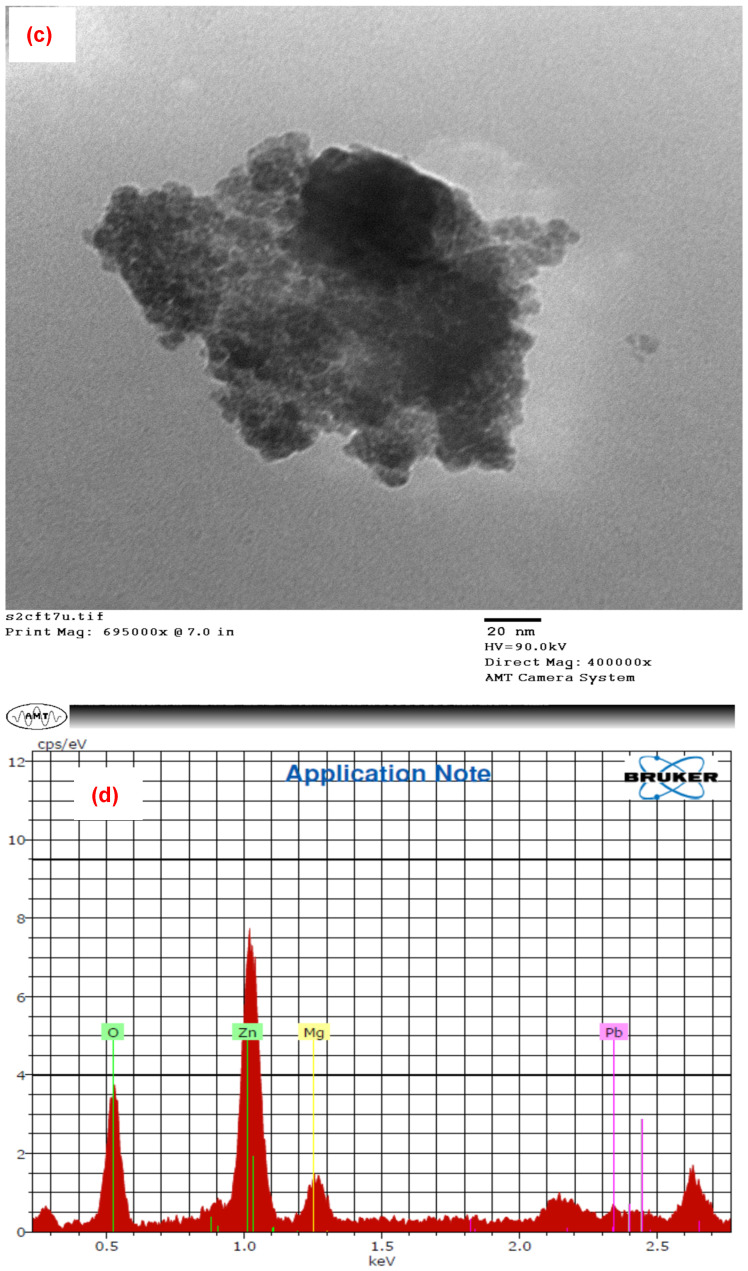

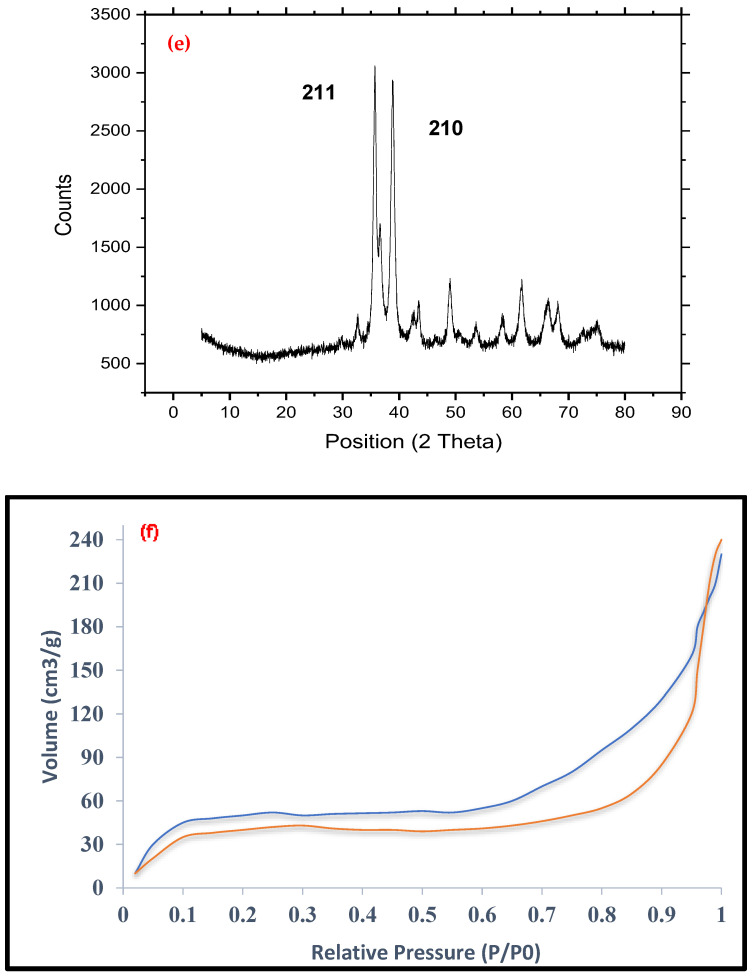
(**a**) SEM image at 400 nm for size, (**b**) SEM image at high low magnification, (**c**) TEM image, (**d**) EDX spectra, (**e**) XRD spectrum, and (**f**) adsorption-desorption isotherm curve for nitrogen of PbO@MgZnO.

The PbO@MgZnO was formed using a co-precipitation method followed by ultrasonication for up to 1.5 h. The synthesized PbO@MgZnO is characterized by FE-SEM, TEM, EDX, XRD, and nitrogen sorption techniques. The average size of the nanoparticle measure was 22.2 nm, as illustrated in the high magnification Figure 1a. The low magnification image (Figure 1b) of SEM revealed that some particles are in unit form and some look big due to the clubbing of small nanoparticles over one another. However, during experiments, it is observed that the particle is easily dispersed in unit form when it is poured into the dye solution. TEM images (Figure 1c) confirm the morphology, size, and clubbing of small nanoparticles observed in FE-SEM images. EDX (Figure 1d) illustrates that the adsorbent is made up of oxide of Pb, Mg, and Zinc, having proper concentration without any impurities. The crystalline nature of XRD was revealed in the peaks at 35°, 38°, 43°, 50°, 52°, 57°, 61°, and 66° for PbO@MgZnO (Figure 1e). The N_2_ adsorption-desorption graph (Figure 1f) depicts the unrestricted mono/multilayer adsorption at the macroporous adsorbent surface and reveals the 128 m^2^/g of the specific surface area of PbO@MgZnO.

### 3.2. Effect of pH Variation, Contact Time, and Adsorbent Dose

**Figure 2:**
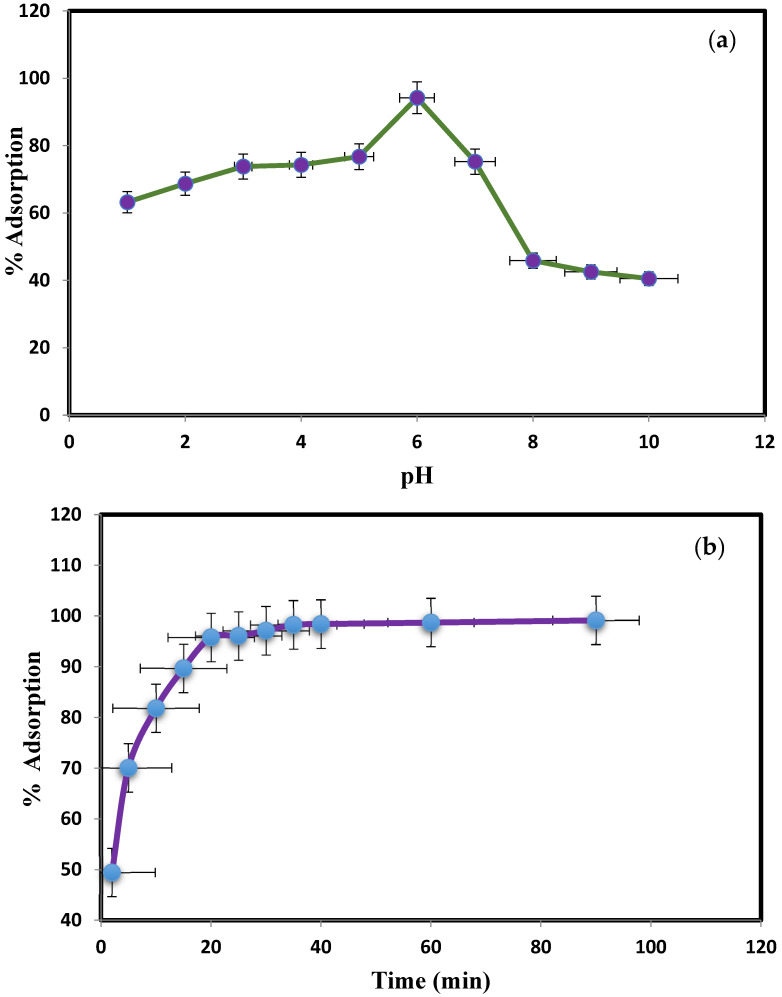

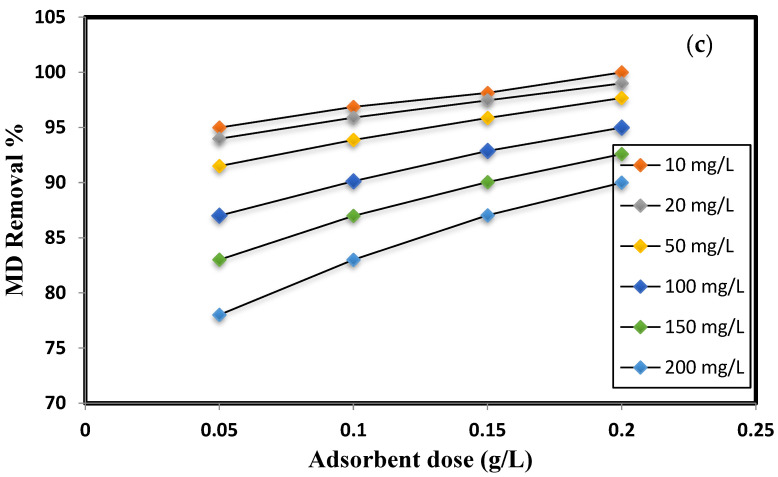
(**a**) MD adsorption changes with pH (PbO@MgZnO = 0.2 g, MD concentration = 50 mg/L, 90 min), (**b**) effect of time on the adsorption of MD dye, and (**c**) variation of PbO@MgZnO dose (at 25 °C).

pH plays a crucial role in the adsorption of dye on nanoparticles. The impact of pH on color adsorption utilizing PbO@MgZnO was assessed at different pH ranges from 1 to 10. Figure 2a illustrates the most notable adsorption of 94.2% at pH 6.0, this is due to the protonation on PbO@MgZnO. MD was protonated and the increment in % adsorption was due to the electrostatic attraction with PbO@MgZnO at this pH. We illustrated the test by changing the time from 0–90 min in Figure 2b, the greatest adsorption of MD was accomplished after 40 min, which was almost 98.4%. This adsorption is obtained since most active sites are accessible, and in this contact time the exchanging of molecules for adsorption interactions was completed, hence 40 min time is sufficient. In batch tests, the impact of PbO@MgZnO dose on MD adsorption was investigated by adding adsorbents in the 0.05–0.2 g range to the 50 mL of dye solution (10 mg/L to 200 mg/L dye concentration). Figure 2c illustrates that the efficiencies of adsorption (%) improved from 91.49 to 97.68% by an increase of 0.05 to 0.20 g adsorbent, owing to the more available adsorption sites.

### 3.3. Rate of Adsorption and Thermodynamic Parameter

**Figure 3:**
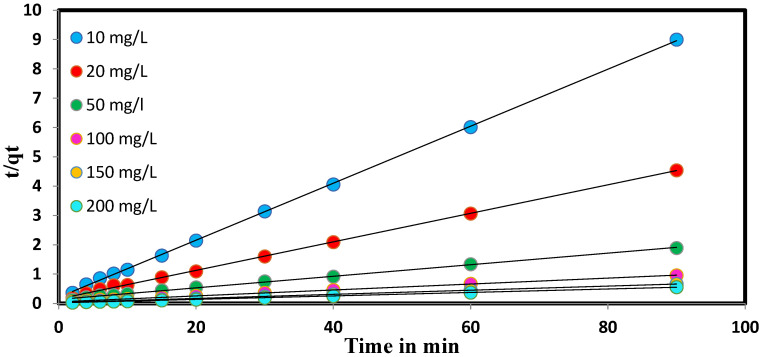
MD kinetic plots on PbO@MgZnO at different initial MD concentrations.

**Figure 4:**
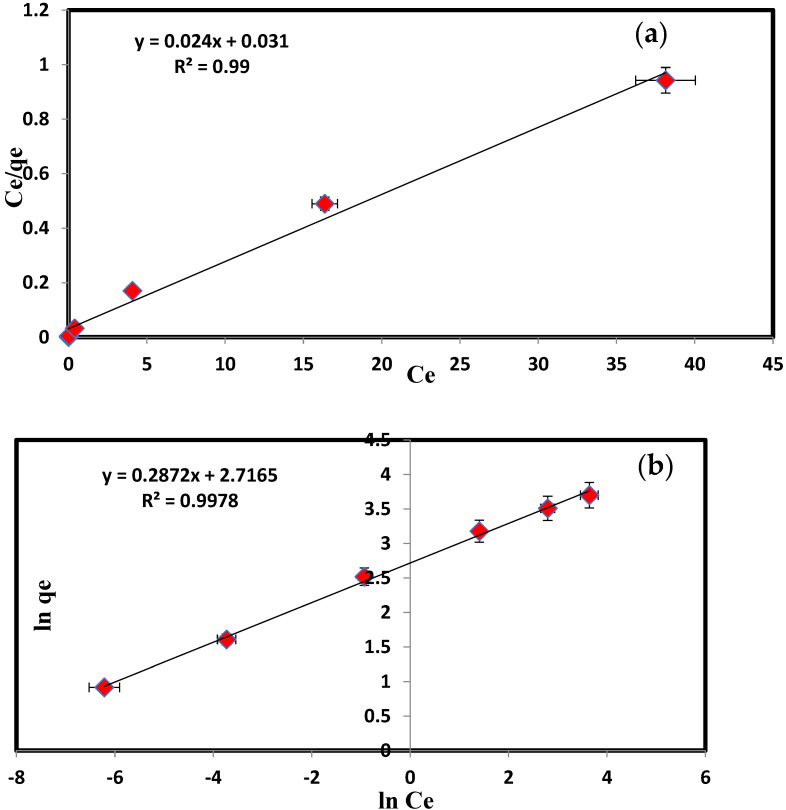

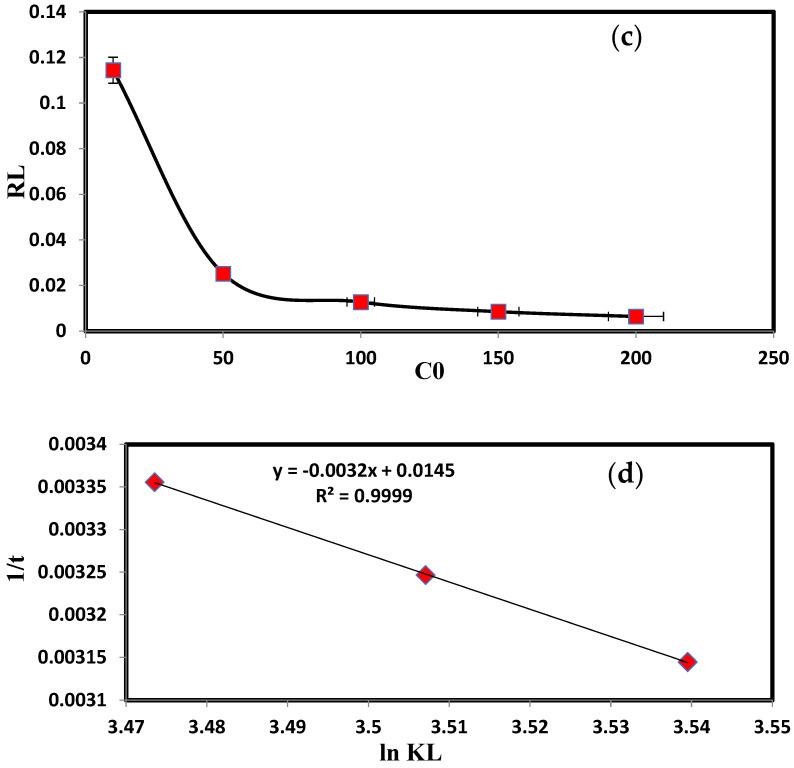
(**a**) Langmuir isotherm graph, (**b**) Freundlich plot for the adsorption, (**c**) separation factor for the adsorption of MD onto PbO@MgZnO, and (**d**) temperature variation graph of the adsorption of MD on PbO@MgZnO.

The energy of MD adsorption on PbO@MgZnO has been analyzed using models suggested by Ho and McKay [44,45,46,47]. The kinetics study affirmed that the adsorption of dye on PbO@MgZnO demonstrates the pseudo-second-order (Figure 3 and Table 1) (Equations are given in Supplement File). Using the famous Langmuir [48,49] and Freundlich [50,51] models, the adsorption of MD was analyzed (Equations are given in Supplement File). Figure 4a,b illustrate isotherms of adsorption, and the measured parameters appeared in Table 2. So, we can conclude that the above study could be a homogenous adsorption of a monolayer, and due to good agreement of the Freundlich model, double and multilayer models can also be followed by the adsorption process. The separation factor (R_L_) is in between 0 to 1 and supports successful adsorption (Figure 4c). The MD dye adsorbed on PbO@MgZnO as a function of the temperature of the system appeared in Figure 4d. The ΔG was determined to be −8605.89 Jmol^−1^. The negative ΔG refers to the spontaneity of MD adsorption on PbO@MgZnO and demonstrates that the adsorption proceeds towards stability [52,53].

## 4. Interpretation of the Steric Parameters

**Figure 5:**
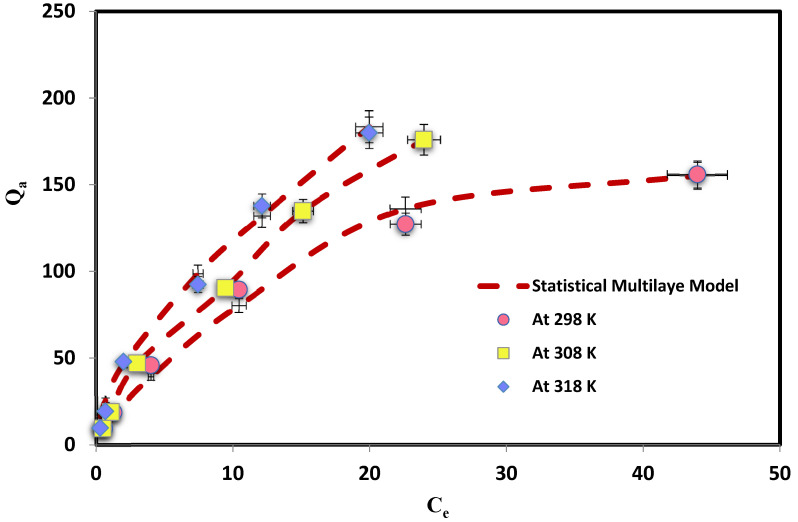
Fitting of mathematical model for the system MD-PbO@MgZnO @ three different temperatures.

**Figure 6:**
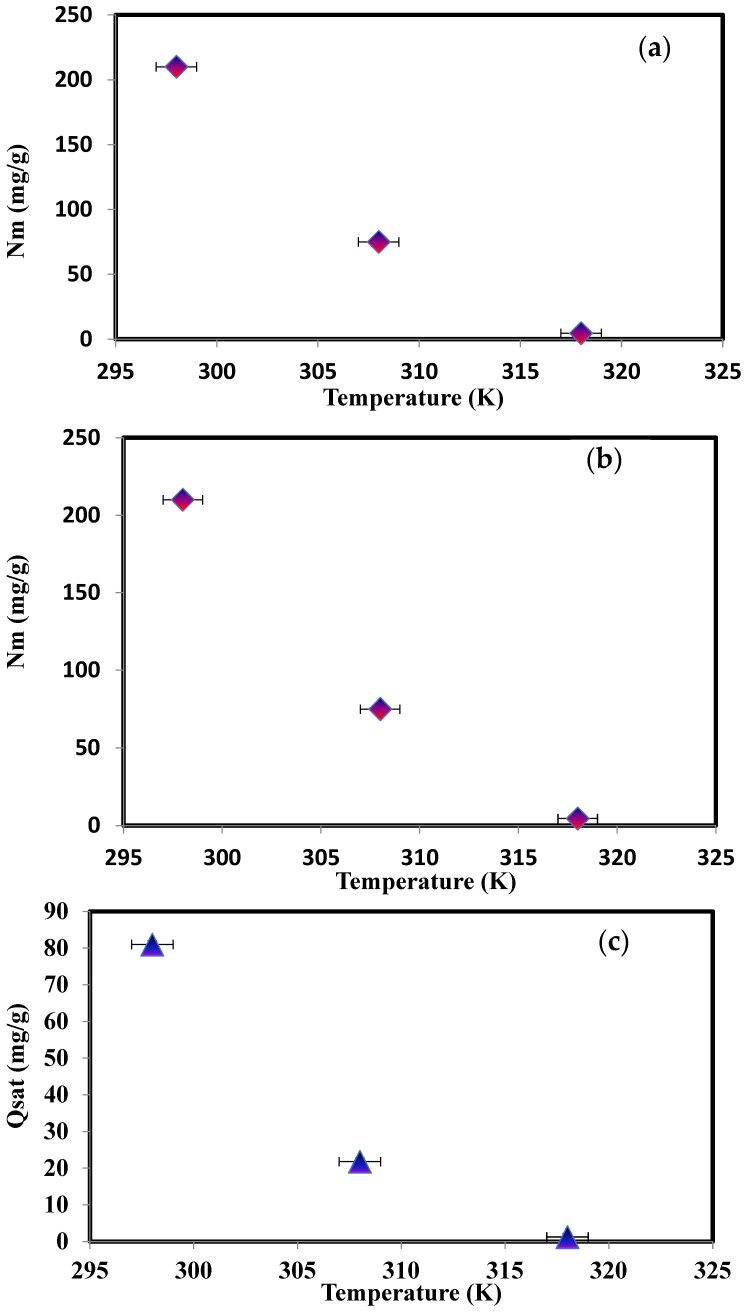

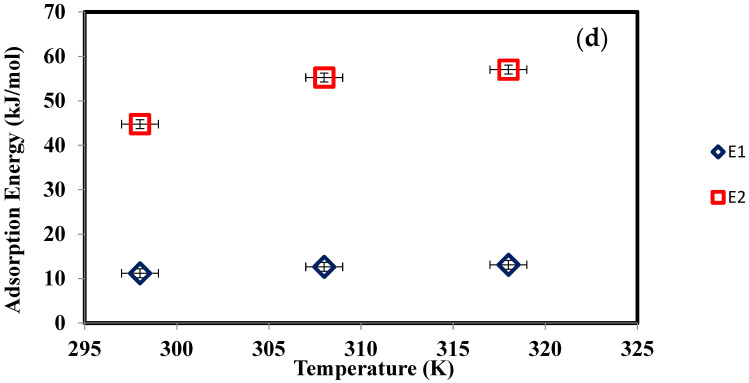
Graph of (**a**) number of molecules per site (*n*), (**b**) Nm, (**c**) Q_sat_, and (**d**) adsorption energies vs. temperature.

An important parameter *n* is the orientation of the MD molecules on the PbO@MgZnO surface (either horizontally or vertically). If *n* is fewer or more than one, this is likely due to the adsorption incorporation of the isolated MD molecules being horizontal or vertical. In addition, multi-docking (*n* < 1) or multimolecular (*n* > 1) can be used to adsorb MD dye on PbO@MgZnO’s (Figure 5). Consequently, an MD molecule can be adsorbed at *n* < 1 on the various active sites of PbO@MgZnO, although, many dye molecules can also be adsorbed at *n* > 1 [54,55,56]. Provided in Figure 6a and Table 3 are the values of the other model parameters, including parameter *n*. *n* = 0.395, 0.290, and 0.280, respectively, at 298, 308, and 318 K (i.e., all values were below 1). MD-to-PbO@MgZnO active-site interactions thus incorporated horizontal molecular positioning and multi-locking processes [55,56]. This finding demonstrates that single MD molécules interacted in the horizontal direction with various active PbO@MgZnO sites and adsorbed MD molecules [55,56]. In general (*n* < 0.5): this scenario revealed that an MD molecule may be interacting with at least two PbO@MgZnO adsorption sites. Then, the best-fitting model is used to derive Nm, Q, and parameters, as indicated in Table 3. The RMSE value of models 1 to model 3 is provided in Table 4; the R^2^ value for model 3 is very high, which demonstrates that model 3 is best fitted for the current adsorption mechanism.

The shift in the PbO@MgZnO active site number (Nm parameter) for the adsorption temperature is illustrated in Figure 6b. For MD adsorption at 298, 308, and 318 K, see Table 3 for Nm values, which were 210, 75, and 4.5 mg/g, respectively. The Nm value decreases due to the *n* parameter decrease. Usually, a decline in the number of PbO@MgZnO functional positions occupied was the product of the adsorption mechanism consistent with the decline of the *n* parameter and, subsequently, the reduction of the Nm parameter. The reduction in the Nm values often indicates the degradation of the contribution of this adsorbent’s new active sites (PbO@MgZnO) to the phase of MD adsorption.

To complement an understanding of the adsorption process, the assessment of complete adsorption layers is necessary [56]. Nt values for MD dye adsorption were estimated at 298, 308, and 318 K are 1.008, 1.001, and 1.0001, (Table 4). During a minor shift in Nt values at all temperatures, the negligible position of this parameter in regulating the adsorption mechanism was established. Therefore, the adsorption function may be omitted from the influence of the parameter N_2_.

Qsat values are determined to classify the efficacy of MD dye adsorption from an aqueous solution using the PbO@MgZnO. The value for Q_sat_ is illustrated in Figure 6c at all temperatures measured, and Table 3 provides a summary of these findings. The value of Qsat at 298, 308, and 318 K, were 81.01, 21.77, and 1.26 mg/g respectively. The low interaction between MD molecules and PbO@MgZnO was confirmed by decreasing Qsat values with temperature. The decrease in solution temperature and adsorption capability can be attributed to the decline of the dye molecules’ mobility, which often precludes the MD’s contact with a wide number of PbO@MgZnO receptor sites [56]. The analysis Table 5 illustrates that PbO@MgZnO can be advised as an efficient adsorbent to treat polluted MD dye from waste water. 

The ε_1_ and ε_2_ present interactions between molecules and the surface of the first and second layers, respectively. We found that both adsorption energies increased concerning temperature, due to the thermal motions. The adsorption energy at 298 K was 44.74 kJ/mol, and the adsorption energy at 318 K was 57.01 kJ/mol (Figure 6d). This increase in energy was explained by the influence of temperature on the surface, which excited the atoms, and which could easily record adsorbed atoms in the surface volume.

## 5. Reuse of PbO@MgZnO

**Figure 7:**
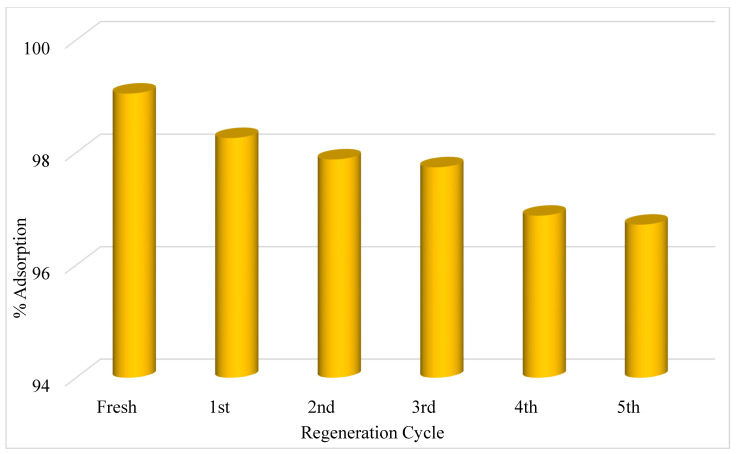
Reuse of PbO@MgZnO for the MD dye.

To investigate the reuse capacity of PbO@MgZnO, the adsorbed color on nanoparticles was desorbed utilizing hydrochloric acid (0.1 M), and PbO@MgZnO was repeatedly reused up to five times. PbO@MgZnO demonstrated a good % adsorption capacity even after reuse five times (Figure 7). The aforesaid study and its findings indicate that PbO@MgZnO is a suitable material for industrial wastewater treatment.

## 6. Conclusions

PbO@MgZnO demonstrates a significant adsorption capability toward Magenta Dye (MD). The greatest adsorption capability was optimized by varying factors such as pH, MD concentration, and adsorbent dose. The negative ΔG refers to the spontaneity of MD adsorption on PbO@MgZnO. The steric parameters from statistical physics models also favor the multilayer adsorption mechanism. At 298, 308, and 318 K, the parameter *n* pattern as a function of solution temperature is *n* = 0.395, 0.290, and 0.280, respectively (i.e., all values were below 1). MD-to-PbO@MgZnO active-site interactions thus incorporated horizontal molecular positioning and multi-locking processes. Mathematical models’ study is also in favor of the multi-layer adsorption phenomenon of MD onto PbO@MgZnO. Therefore, this multifunctional nanocomposite could be used as an adsorbent as well.

## Figures and Tables

**Table 1:** Kinetic Model parameters.

Kinetic Model Parameters						
Concentration (ppm)	10	20	50	100	150	200
K_ad_gmg^−1^ min^−1^	0.0415	0.0136	0.0026	0.0018	0.0012	0.0013
qe (mg/g)	10.64	21.74	52.63	100.00	166.67	333.33
R^2^	0.999	0.999	0.998	0.998	0.999	0.999

**Table 2:** Data from isotherm.

Langmuir		Freundlich	
K_L_	32.25	log K_f_	2.176
q_max_ = K_L_/α_L_	333.33	1/*n*	0.287
R^2^	0.99	R^2^	0.997

**Table 3:** Summary of parameters of the statistical model obtained from MD adsorption onto PbO@MgZnO.

Temperature (K)	N_m_ (mg/g)	*n*	N_t_ = 1 + N_2_	ε_1_ (kJ/mol)	ε_2_ (kJ/mol)	Q_sat_ (mg/g)
298	210	0.385	1.002	11.195	44.744	81.011
308	75	0.290	1.001	12.643	55.225	21.771
318	4.5	0.280	1.0001	13.107	57.018	1.260

**Table 4:** RMSE and R^2^ values for the under-study models for the system of MD on PbO@MgZnO.

Temperature	298		308		318	
(K)	R^2^	RMSE	R^2^	RMSE	R^2^	RMSE
Model 1	0.988	0.6168	0.995	1.8782	0.992	9.094
Model 2	0.983	0.6899	0.996	1.3724	0.994	0.836
Model 3	0.997	1.4534	0.998	5.415	0.998	4.157

**Table 5:** Comparison of adsorption capacity of PbO@MgZnO with other adsorbents.

Adsorbent	Q_max_ (mg/g)	Reference
Thorn apple leaf powder	1.059	[57]
Graphene oxide modified sugarcane bagasse	145	[58]
H_2_SO_4_ activated immature Gossypium Hirsutum seeds	86.24	[59]
Gracilaria edulis algae	1250	[60]
Lyngbya wollei algae	333	[60]
Fe/Cu nanocomposites	235	[61]
Natural Clay	198	[62]
PbO@MgZnO	333	Present study

## Data Availability

The data will be share on request.

## Supplementary Materials

The following supporting information can be downloaded at: https://www.mdpi.com/article/10.3390/ijerph191912199/s1. References [63,64,65,66,67,68,69,70,71,72,73,74] are cited in the supplementary materials.
