# Supplementary material for: Adsorption of Magenta Dye on PbO Doped MgZnO: Interpretation of Statistical Physics Parameters Using Double-Layer Models
Source: Int J Environ Res Public Health. 2022 Sep 26;19(19):12199. doi: 10.3390/ijerph191912199 (PMC9564486; doi:10.3390/ijerph191912199)
Supplement: Supplementary file 1 [file ijerph-19-12199-s001.zip › ijerph-1858635-supplementary.pdf]

Adsorption of Magenta Dye on PbO doped MgZnO: Interpretation of Statistical Physics Parameters using Double-layer Models

Tariq Altalhi[a], Ganesh Jethave[b], Umesh Fegade*[c], Gaber A. M. Mersal[a], Mohamed M. Ibrahim[a], M.H.H. Mahmoud[a], Tushar Kumeria[d], Kalpesh A. Isai[e], Milind Sonawane[e]

[a]Department of Chemistry, College of Science, Taif University, P.O. Box 11099, Taif 21944, Saudi Arabia
[b]Department of Chemistry, Dr. Annasaheb G. D. Bendale Mahila Mahavidyalaya, Jalgaon 425001
[c]Department of Chemistry, Bhusawal Arts, Science and P. O. Nahata Commerce College, Bhusawal, M. S. India.
[d]School of Materials Science and Engineering, The University of New South Wales, Sydney, New South Wales, 2052, Australia
[e]Department of Applied Science and Humanities, R. C. Patel Institute of Technology, Shirpur (MH), India
Corresponding author *Email ID: umeshfegade@gmail.com (U. F)

Supplementary Data

1. Chemical kinetic models (Pseudo-second-order)

The kinetics of MD adsorption on PbO@MgZnO was investigated by the Ho and McKay model; it is common used model that describes kinetics of dye adsorption on solid nanoparticles. The pseudo-second order model, proposed by Ho and McKay, can be represented in the following linear form [1].

In this model, graph was plotted in between t/qt and t and experimental data was determined which show its appropriateness for explanation of whole adsorption process [2]. It was found that the plot of t/qt versus t is a linear straight line.

The pseudo–second–order kinetic model is described by the formula

$$t/q_t = 1/k_{ad}\, q_e^2 + t/q_e \quad \text{--- Eq. S1}$$

The results showed that the adsorption system followed the Ho and McKay equations for the entire adsorption.

2. Adsorption isotherms

The adsorption isotherms play an important role in determining the adsorption skill of MD onto PbO@MgZnO nanoparticles. Langmuir isotherm does not take into depiction the variation in adsorption energy, but it is the easiest explanation of the adsorption process [4]. It is used for the description of monolayer adsorption with interaction between adsorbed molecules [5, 6].

The general equation of the Langmuir isotherm [3] is:

$$q_e \ \alpha_L/K_L = K_L C_e/(1+K_L C_e) \qquad \text{Eq. S2}$$

After linearization of the Langmuir isotherm, Eq. (S3), we obtain

$$C_e/q_e = C_e\alpha_L/K_L + 1/K_L \qquad \text{Eq. S3}$$

where Ce is the equilibrium concentration and qe is the amount of MD dye adsorbed in the solution (mg/L), per unit mass of adsorbent (mg/g), at equilibrium concentration, Ce, α_L (Lmg^{-1}) and K_L ($L\ g^{-1}$) are the Langmuir constants with αL related to the adsorption energy and q_{max} [$=K_L/\alpha_L$] signifies the maximum adsorption capacity (mg/g), which depends on the number of adsorption sites. The Freundlich equilibrium isotherm equation was also used to describe experimental adsorption data [7].

The linearized form of the Freundlich adsorption isotherm equation is

$$\ln q_e = \ln K_f + \ln C_e/nf \qquad \text{Eq. S4}$$

where Ce is the equilibrium concentration (mg/L), qe is the amount of dye adsorbed at equilibrium (mg/g), Kf (mg^{1-n} L^n g^{-1}) and 1/n are Freundlich constants depending on the temperature and the given adsorbent–adsorbate couple [8]. The values of Kf and 1/n calculated from the intercept and slope of the plot of ln qe Vs. ln Ce.

3. Separation Factor

The Langmuir isotherm can be expressed in terms of a dimensionless constant separation factor (R_L) given by the following equation:

$$R_L = 1/\ (1+\alpha_L\ C_o) \qquad \text{-- Eq. S5}$$

R_L values within the range $0 < R_L < 1$ indicate favorable adsorption [9,10]. In this study, R_L value of PbO@MgZnO for the initial MD concentration of 2650 mg/L, obtained as 0.583, indicate favorable adsorption of MD onto them.

4. Adsorption Thermodynamic parameters

Thermodynamic parameters can offer further comprehensively information concerning the intrinsic energetic changes in adsorption process [11]. The G value indicates the nature of the reaction whether the reaction is spontaneous or nonspontaneous [12].

$$\Delta G^\circ = RT \ln K_L \qquad \text{-- Eq. S6}$$

References:

1. Das, P., & Debnath, A. (2021). Reactive orange 12 dye adsorption onto magnetically separable CaFe2O4 nanoparticles synthesized by simple chemical route: kinetic, isotherm and neural network modeling. *Water Practice & Technology*, *16*(4), 1141-1158.

2. Hussain, S., Kamran, M., Khan, S.A., Shaheen, K., Shah, Z., Suo, H., Khan, Q., Shah, A.B., Rehman, W.U., Al-Ghamdi, Y.O. and Ghani, U., (2021). Adsorption, kinetics and thermodynamics studies of methyl orange dye sequestration through chitosan composites films. *International journal of biological macromolecules*, *168*, 383-394.
3. Hii, H. T. (2021). Adsorption isotherm and kinetic models for removal of methyl orange and remazol brilliant blue r by coconut shell activated carbon. *Tropical Aquatic and Soil Pollution*, *1*(1), 1-10.
4. Aniagor, C. O., Afifi, M. A., & Hashem, A. (2021). Modelling of basic blue-9 dye sorption onto hydrolyzed polyacrylonitrile grafted starch composite. *Carbohydrate Polymer Technologies and Applications*, *2*, 100141.
5. Grabi, H., Lemlikchi, W., Derridj, F., Lemlikchi, S., & Trari, M. (2021). Efficient native biosorbent derived from agricultural waste precursor for anionic dye adsorption in synthetic wastewater. *Biomass Conversion and Biorefinery*, 1-18.
6. Oueslati, K., Naifar, A., Sakly, A., Kyzas, G. Z., & Lamine, A. B. (2022). Statistical and physical interpretation of dye adsorption onto low-cost biomass by using simulation methods. *Colloids and Surfaces A: Physicochemical and Engineering Aspects*, *646*, 128969.
7. Elsherif, K., El-Dali, A., Alkarewi, A., Ewlad-Ahmed, A., & Treban, A. (2021). Adsorption of crystal violet dye onto olive leaves powder: Equilibrium and kinetic studies. *KM Elsherif, A. El-Dali, AA Alkarewi, AM Ewlad-Ahmed and A. Treban. Adsorption of crystal violet dye onto olive leaves powder: Equilibrium and kinetic studies. Chemistry International*, *7*(2), 79-89.

8. Raj, A., Yadav, A., Rawat, A.P., Singh, A.K., Kumar, S., Pandey, A.K., Sirohi, R. and Pandey, A., (2021). Kinetic and thermodynamic investigations of sewage sludge biochar in removal of Remazol Brilliant Blue R dye from aqueous solution and evaluation of residual dyes cytotoxicity. *Environmental Technology & Innovation*, *23*, 101556.
9. Bensalah, J., Habsaoui, A., Dagdag, O., Lebkiri, A., Ismi, I., Rifi, E.H., Warad, I. and Zarrouk, A., (2021). Adsorption of a cationic dye (Safranin) by artificial cationic resins AmberliteŪIRC-50: Equilibrium, kinetic and thermodynamic study. *Chemical Data Collections*, *35*, 100756.
10. Yusuff, A. S., Ajayi, O. A., & Popoola, L. T. (2021). Application of Taguchi design approach to parametric optimization of adsorption of crystal violet dye by activated carbon from poultry litter. *Scientific African*, *13*, e00850.
11. Sharma, K., Sharma, S., Sharma, V., Mishra, P. K., Ekielski, A., Sharma, V., & Kumar, V. (2021). Methylene blue dye adsorption from wastewater using hydroxyapatite/gold nanocomposite: Kinetic and thermodynamics studies. *Nanomaterials*, *11*(6), 1403.
12. Banisheykholeslami, F., Hosseini, M., & Darzi, G. N. (2021). Design of PAMAM grafted chitosan dendrimers biosorbent for removal of anionic dyes: adsorption isotherms, kinetics and thermodynamics studies. *International Journal of Biological Macromolecules*, *177*, 306-316.
